# Test–retest reliability of the EQ-5D-5L and the reworded QOLIBRI-OS in the general population of Italy, the Netherlands, and the United Kingdom

**DOI:** 10.1007/s11136-021-02893-3

**Published:** 2021-06-01

**Authors:** Di Long, Suzanne Polinder, Gouke J. Bonsel, Juanita A. Haagsma

**Affiliations:** 1 Department of Public Health, Erasmus MC, University Medical Center Rotterdam, P.O. Box 2040, 3000 CA Rotterdam, The Netherlands; 2 EuroQol Group Executive Office, Rotterdam, The Netherlands

**Keywords:** Test–retest reliability, Web-based, EQ-5D-5L, Reworded QOLIBRI-OS

## Abstract

**Purpose:**

To assess the test–retest reliability of the EQ-5D-5L and the reworded Quality of Life After Traumatic Brain Injury Overall Scale (QOLIBRI-OS) for the general population of Italy, the Netherlands, and the United Kingdom (UK).

**Methods:**

The sample contains 1864 members of the general population (aged 18–75 years) of Italy, the Netherlands, and the UK who completed a web-based questionnaire at two consecutive time points. The survey included items on gender, age, level of education, occupational status, household annual income, chronic health status, and the EQ-5D-5L and reworded QOLIBRI-OS instrument. Test–retest reliability of the EQ-5D-5L dimensions, EQ-5D-5L summary index, EQ VAS, reworded QOLIBRI-OS dimensions and reworded QOLIBRI-OS level sum score was examined by Gwet’s Agreement Coefficient (Gwet’s AC) and Intraclass Correlation Coefficient (ICC).

**Results:**

Gwet’s AC ranged from 0.64 to 0.97 for EQ-5D-5L dimensions. The ICC ranged from 0.73 to 0.84 for the EQ-5D-5L summary index and 0.61 to 0.68 for EQ VAS in the three countries. Gwet’s AC ranged from 0.35 to 0.55 for reworded QOLIBRI-OS dimensions in the three countries. The ICC ranged from 0.69 to 0.77 for reworded QOLIBRI-OS level sum score.

**Conclusion:**

Test–retest reliability of the EQ-5D-5L administered via a web-based questionnaire was substantial to almost perfect for the EQ-5D-5L dimensions, good for EQ-5D-5L summary index, and moderate for the EQ VAS. However, test–retest reliability was less satisfactory for the reworded QOLIBRI-OS. This indicates that the web-based EQ-5D-5L is a reliable instrument for the general population, but further research of the reworded QOLIBRI-OS is required.

**Supplementary Information:**

The online version of this article (10.1007/s11136-021-02893-3) contains supplementary material, which is available to authorized users.

## Introduction

Health-related quality of life (HRQoL) questionnaires serve the purpose of assessing an individual's or a group's perceived physical and mental health and may be used to assess population health [1], develop policy [2], and evaluate health programs [3]. HRQoL questionnaires can be classified as generic or disease-specific. The EQ-5D is one of the most commonly used generic instruments to measure HRQoL [4]. The five-level version EQ-5D-5L is based on a self-reported health status that consists of five dimensions with the range of responses to each dimension being five levels, together with a visual analogue scale (EQ VAS). The scores from the EQ-5D dimensions can be converted into an index score by applying health preference weights elicited from a general population.

The Quality of Life After Traumatic Brain Injury Overall Scale (QOLIBRI-OS) is a short 6-item version of the 37-item QOLIBRI questionnaire [5]. The complete QOLIBRI is a disease-specific HRQoL instrument for patients with Traumatic Brain Injury (TBI); the OS-component measures overall satisfaction with different aspects of health. After removing references to TBI in the instructions, for example, “How satisfied are you with your physical condition (instead of “*since your brain injury*”)?”, the reworded QOLIBRI-OS can be regarded as a generic instrument suitable for any disease or the general population [6].

The validity and reliability of an HRQoL instrument are essential for evidence-based medical interventions [7]. Test–retest reliability is the major reliability aspect and is defined as the consistency between measurements at two time points. It is based on the assumption that no memory effect nor true changes occur between the measurements [8]. Test–retest reliability of the EQ-5D-5L in the general population, as established by in-person interviews, is good [9–11]. Good reliability of the QOLIBRI-OS in TBI patients has been confirmed [10]. However, the reliability of the reworded QOLIBRI-OS has not yet been measured in the general population. In this paper, both the EQ-5D-5L and the reworded QOLIBRI-OS were offered through web-based questionnaires [12], which offer advantages such as improved data quality [13] and reduction on cost [14]. Disadvantages are uncertainty on the respondents and the risk of drop-out [15, 16]. Neither the reliability of the web-based EQ-5D-5L nor that of reworded QOLIBRI-OS has been established.

This study aimed to assess the test–retest reliability of the web-based versions of the EQ-5D-5L and the reworded QOLIBRI-OS in the general population of Italy, the Netherlands, and the UK.

## Data and methods

### Samples and data collection

The sample consists of 1864 persons of Italy, the Netherlands, and the UK. The data were collected as part of the Collaborative European Neuro Trauma Effectiveness Research in Traumatic Brain Injury (CENTER-TBI) study [17]. The participants were recruited by an international market research agency from existing large internet panels.

The data were collected at two time points within 6 months. At the first time point (T1), a sample that represented the general population by age (18–75), gender, and education in the selected countries, was asked to fill out the web-based questionnaire (from 29th June till 31st July 2017). At the second time point (T2), a random subset of the T1 sample respondents was re-contacted (from 3rd until16th February 2018).

Panel participation was on a volunteer basis, once participating the data capture system did not allow skipping questions. Participants received an incentive in the form of cash or points from the market research company.

### Health instruments

#### EQ-5D-5L

The EQ-5D-5L consists of five dimensions: (having problems in) Mobility, Self-care, Usual activities, Pain/Discomfort, and Anxiety/Depression. The ordinal response options of the 5-level version range from “no problems” (“1”) to extreme problems/unable to (“5”). The EQ-5D-5L profile is the 5-digit combination of dimension responses. It ranges from “11,111” (best health) to “55,555” (worst health).

#### EQ-5D-5L summary index

The EQ-5D-5L summary index is calculated to present an overall health state and to allow comparison between literatures. The EQ-5D summary is a summary of all five dimensions weighted by a value set. The value set consists of weights that can convert each EQ-5D health profile into a single value. The value set is measured by social preferences in a given country, where different weights are attached to each level of each dimension of the EQ-5D [4, 18]. The EQ-5D-5L summary index ranges from below 0 (“worse than dead”) to 1 (“full health”). The EQ-5D-5L summary index for the Netherlands and the UK was calculated using the value set for the Netherlands and the UK, respectively. An EQ-5D-5L value set for Italy is not available. We used the value set for the UK instead.

#### EQ VAS

The EQ VAS asks the respondent to rate their health from 0 to 100 on a visual analogue scale, where 0 is the worst imaginable health state, and 100 is the best imaginable health state.

#### Reworded QOLIBRI-OS

The reworded QOLIBRI-OS has six items: (satisfaction with) Physical condition; Cognition, Emotion, Function in daily life, Personal and social life, and Current situation and future prospects. As with EQ-5D-5L, the response consists of 5 ordinal levels, ranging from “not at all satisfied” (“1”) to “very satisfied” (“5”). The reworded QOLIBRI-OS profile is the 6-digit combination of item responses. It ranges from “555,555” (very satisfied with health) to “111,111” (not satisfied at all with health). The reworded QOLIBRI-OS level sum score is the unweighted summary of the scores on the six dimensions. It can be used as a crude measure of satisfaction to estimate the validity obtained in valuation for studies for different health states. The reworded QOLIBRI-OS ranges from 6 (“not at all satisfied”) to 30 (“very satisfied”) and is treated as a continuous variable.

#### Background variables

The questionnaires contained standard items on age, gender, the highest level of education achieved, occupational status, household annual income, chronic health status, the EQ-5D-5L, and the reworded QOLIBRI-OS.

Level of education was measured as the highest level achieved and coded based on the International Standard Classification of Education (ISCED-97) into three groups: Up to lower secondary education (ISCED 0, 1 and 2; ‘low’), completed upper secondary education (ISCED 3 and 4; ‘mid') and tertiary education (ISCED 5 and 6; ‘high').

Occupational status was grouped into four categories: employed, unemployed (including caregiver and student), retired, and unable to work. Household income was grouped into 4 categories: “Low” (less than €20,000/£14,000), “Middle” (€20,000–€59,999/£14,000–£41.999), and “High” (€60,000/£42,000 and/or more).

Chronic health status was measured by the presence of 12 chronic health conditions (asthma and chronic bronchitis, severe heart disease, stroke, diabetes, severe back complaints, arthrosis, rheumatism, cancer, memory problems due to a neurological condition like dementia, memory problems due to aging, depression, or other problems). For this question, multiple answers were allowed.

### Data-analysis

We selected respondents who experienced no change of chronic conditions between T1 and T2 and assumed that represented no real changes in their health state.

Spearman correlation was used to test the correlation between reworded QOLIBRI-OS items and EQ-5D-5L dimensions. It was hypothesized that similar domains measuring similar items of HRQoL should be strongly correlated (> 0.5) [19].

Bland–Altman (BA) plots were used to visually examine the test–retest agreements for EQ-5D-5L summary index, the EQ VAS, and reworded QOLIBRI-OS level sum score. A BA plot shows whether there are significant systematic differences between T1 and T2, for example, whether the second measurement constantly under- or overestimates compared to the first one.

Test–retest reliability of both EQ-5D-5L and reworded QOLIBRI-OS dimensions/items scores were calculated using Gwet’s AC2 test. Gwet’s AC, proposed by Gwet [20] corrects the chance-agreement probability so that it is consistent with the inclination of random rating from the observed rating. Gwet’s AC1 is for use with nominal data and Gwet’s AC2 is for use with ordinal data. Gwet’s AC addresses the paradoxical behavior of Cohen’s Kappa where low Kappa is coupled with a high agreement. Because Kappa cannot reflect real agreement with very high (or low) trait prevalence [21, 22]. Health data of the general population typically are extremely unbalanced with a high prevalence of traits such as “no problem”. Radical and linear weights are selected to calculate Gwet’s AC2 for EQ-5D-5L and reworded QOLIBRI-OS dimensions/items [23]. We consider Gwet’s AC2 higher than 0.8, between 0.8 and 0.6, between 0.6 and 0.4, between 0.4 and 0.2, and lower than 0.2, to be indicative of almost perfect, substantial, moderate, fair, and slight to none agreement [24]. The percentage of agreement between test and retest was also calculated.

Test–retest reliability of the EQ-5D-5L summary index, the EQ VAS, and the reworded QOLIBRI-OS level sum score were calculated by Intraclass Correlation Coefficient (ICC, two-way random effects, absolute agreement). Because ICC prefers data normality and stable variance [25, 26], our data were transformed using Tukey’s “Ladder of Powers” to acquire near-normality [27]. As an alternative to ICC, Lin’s Concordance Correlation Coefficient (CCC) was calculated to approximate data normality [28]. We consider ICC and CCC values higher than 0.9, between 0.9 and 0.75, between 0.75 and 0.5, and lower than 0.5 are respectively indicative of excellent, good, moderate, and poor reliability [29].

*Z* test [30] after Fisher’s r-to-z transformation was used to compare Gwet’s AC, ICC, and CCC between countries. The significant level was set at 0.05.

All statistical analyses were carried out using R version 3.6.3.

## Results

### Sample

1864 respondents completed the T1 and T2 questionnaire, of which 1171 (62.8%) respondents (IT: 377, NL: 390, UK: 404) reported no change of health status and were included in the analysis.

Of these 1171 respondents, 71 people did not have corresponding answers with regards to gender at T1 and T2, had more than two years difference of age, and/or level of education backward from T1 to T2. Twenty-two respondents spent less than a minute to finish the entire questionnaire, which we consider unrealistically short. Chi-square tests between the 93 and the rest of respondents in each instrument suggest no significant difference in the distribution of EQ-5D-5L and reworded QOLIBRI-OS domains (Supplementary file 1), we thus did not exclude these 93 respondents in our analysis and assumed that information at T1 is correct. Characteristics of the respondents at T1 are shown in Table 1. There are significant differences between countries in the distribution of age, level of education, occupational status, and level of income.

**Table 1:** Characteristics of the respondents at T1 by country

	Italy (*N* = 377)	Netherlands (*N* = 390)	UK (*N* = 404)
	*N* (%)	*N* (%)	*N* (%)
Age			
Median (Q1, Q3)	43 (32.0, 55.0)	47 (36.0, 57.0)	44 (34.0, 56.2)
Gender			
Male	205 (54.4%)	206 (52.8%)	206 (51.0%)
Female	172 (45.6%)	184 (47.2%)	198 (49.0%)
Education level			
Low	97 (25.7%)	92 (23.6%)	94 (23.3%)
Middle	226 (59.9%)	204 (52.3%)	182 (45.0%)
High	54 (14.3%)	94 (24.1%)	128 (31.7%)
Occupational status			
Employed	178 (47.2%)	224 (57.4%)	227 (56.2%)
Unemployed	143 (37.9%)	76 (19.5%)	79 (19.6%)
Retired	54 (14.3%)	51 (13.1%)	68 (16.8%)
Unable to work	2 (0.5%)	39 (10.0%)	30 (7.4%)
Household income level			
Low	92 (24.4%)	69 (17.7%)	77 (19.1%)
Middle	203 (53.8%)	187 (47.9%)	216 (53.5%)
High	29 (7.7%)	38 (9.7%)	83 (20.5%)
Unknown	53 (14.1%)	96 (24.6%)	28 (6.9%)

### HRQoL

**Fig. 1:**
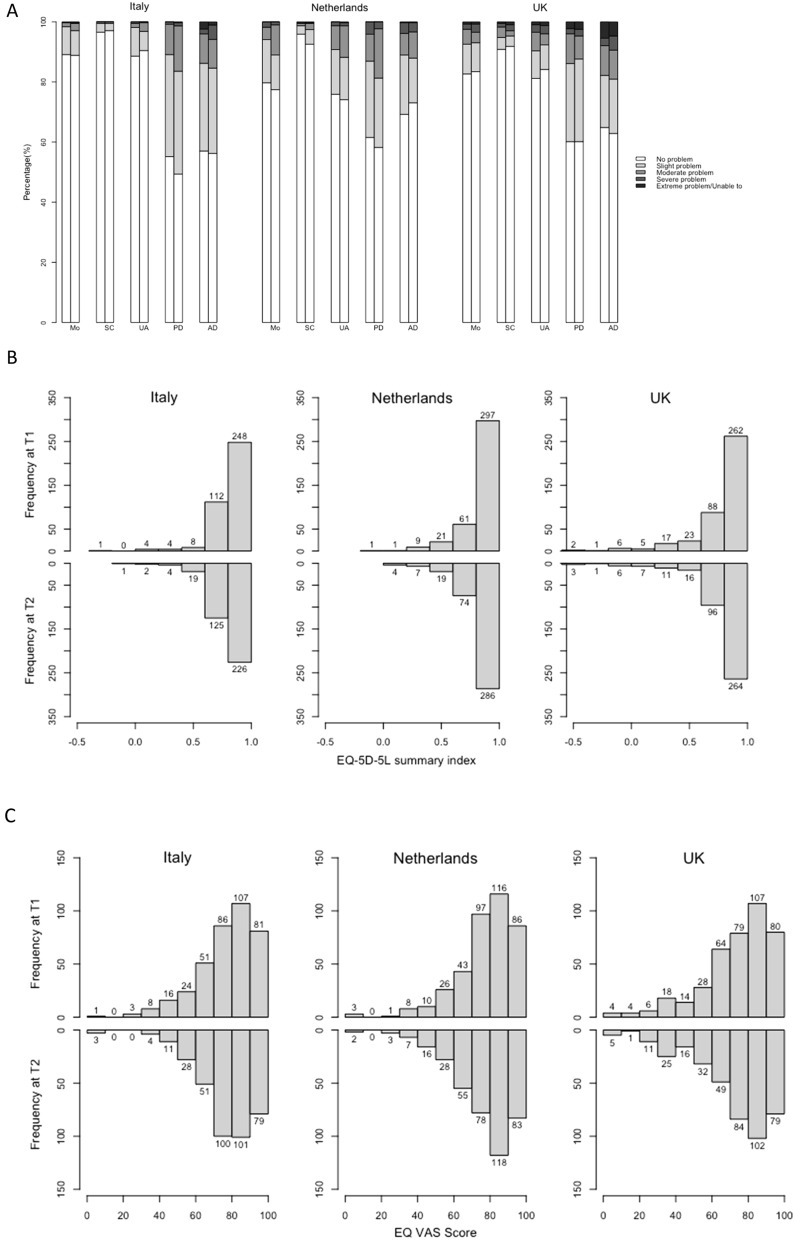

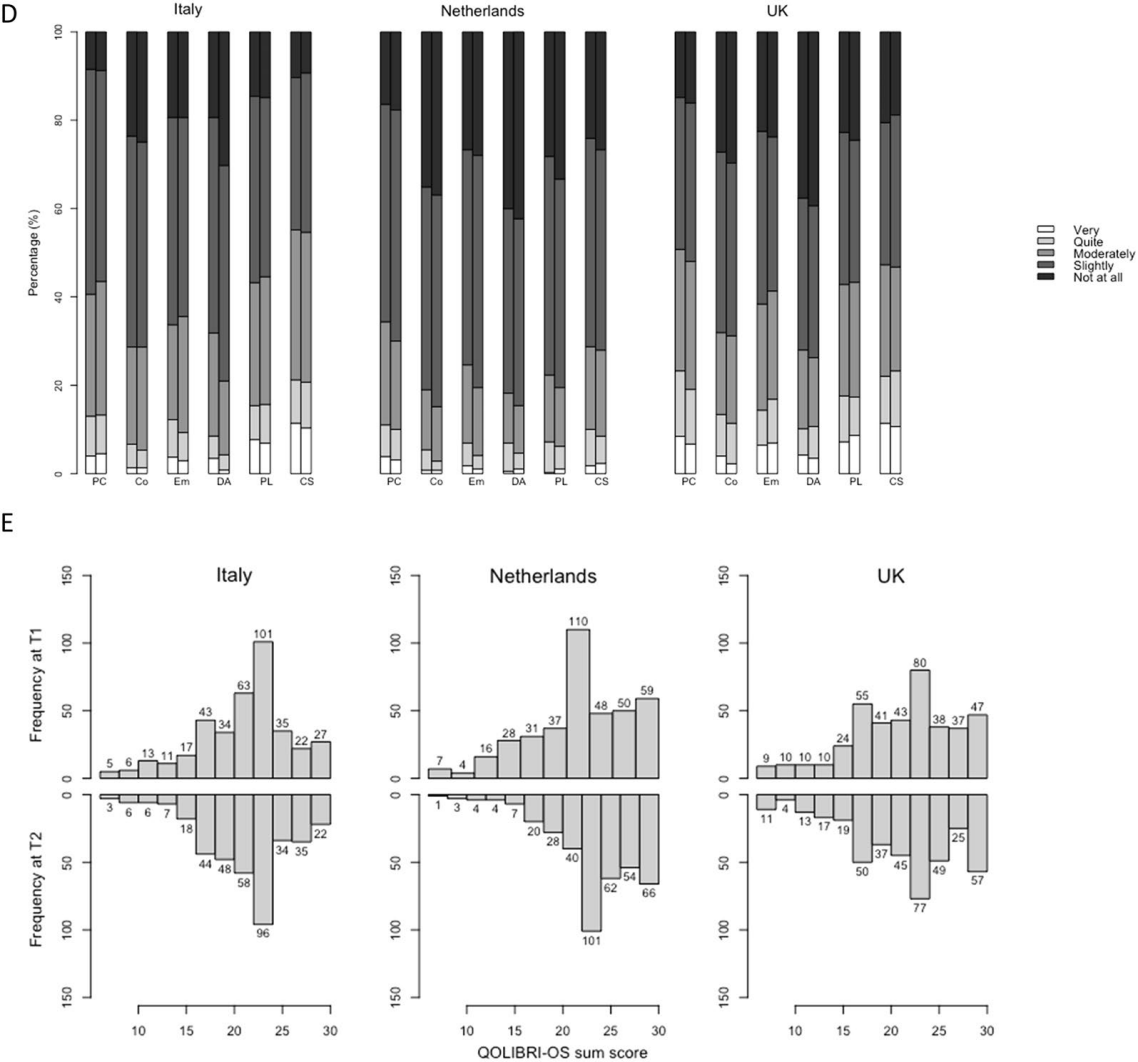
(Cross-sectional) Distribution of the EQ-5D-5L dimensions (**a**), the EQ-5D-5L summary index (**b**), the EQ VAS (**c**), the reworded QOLIBRI-OS items (**d**), and the reworded QOLIBRI-OS level sum score (**e**) in T1 and T2 in Italy, the Netherlands, and the UK. Note to figure: in Fig. 1a, abbreviations Mo, SC, UA, PD, and AD refer to EQ-5D-5L dimensions Mobility, Self-care, Usual activities, Pain/Discomfort and Anxiety/Depression, respectively. In Fig. 1d, abbreviations PC, Co, DA, PL, and CS refer to reworded QOLIBRI-OS items Physical condition, Cognition, Emotion, Function in daily life, Personal and social life, and Current situation and future prospects, respectively

Summary distributions of the EQ-5D-5L dimensions, the EQ VAS, the EQ-5D-5L summary index, and the reworded QOLIBRI-OS items are shown in Fig. 1. Overall, 38%, 46%, and 47% of respondents respectively in Italy, the Netherlands, and the UK reported a perfect health state (profile “11,111” for EQ-5D-5L) at T1; at T2 the correspondent percentages were 35%, 48%, and 44%. Median (IQR) EQ-5D-5L summary index scores were 0.88(0.23), 0.89(0.19), 0.88(0.23) respectively in Italy, the Netherlands, and the UK at T1, and 0.84(0.23), 0.91(0.21), 0.85(0.23) respectively at T2. 63%, 63%, and 54% of respondents, respectively in Italy, the Netherlands, and the UK reported EQ VAS higher than 80 at T1, at T2 the correspondent percentage were 62%, 59%, and 55%. Median EQ VAS were 80, 81, and 80, respectively in Italy, the Netherlands, and the UK at both T1 and T2.

With regard to the reworded QOLIBRI-OS, less than 10% of the respondents in each country reported being very satisfied with their health (“555,555”) at both T1 and T2 (5%, 8%, and 8%, respectively, in Italy, the Netherlands, and the UK at T1; 4%, 10%, and 9% at T2). Median (IQR) reworded QOLIBRI-OS level sum score was 22.0(6), 24.0(6), 22.5(8), respectively in Italy, the Netherlands, and the UK at T1, and 22.0(5), 24.0(5), 23.0(8) at T2, respectively.

Among all respondents with a perfect EQ-5D-5L health state (“11,111”), less than 20% responded being very satisfied on the reworded QOLIBRI-OS (15% at T1 and 17% at T2). Spearman correlation showed strong correlations between EQ-5D-5L index and reworded QOLIBRI-OS level sum score (0.52 at T1 and 0.53 at T2) and between domain “Anxiety/Depression” of the EQ-5D-5L and “Emotion” of the reworded QOLIBRI-OS (0.51 at both T1 and T2.). However, for the domains that are most comparable between the two instruments (“Usual activities” of the EQ-5D-5L and “Function in daily life” of the reworded QOLIBRI-OS)—the Spearman correlation did not show strong correlations between the responses on these domains (0.45 at both T1 and T2).

**Fig. 2:**
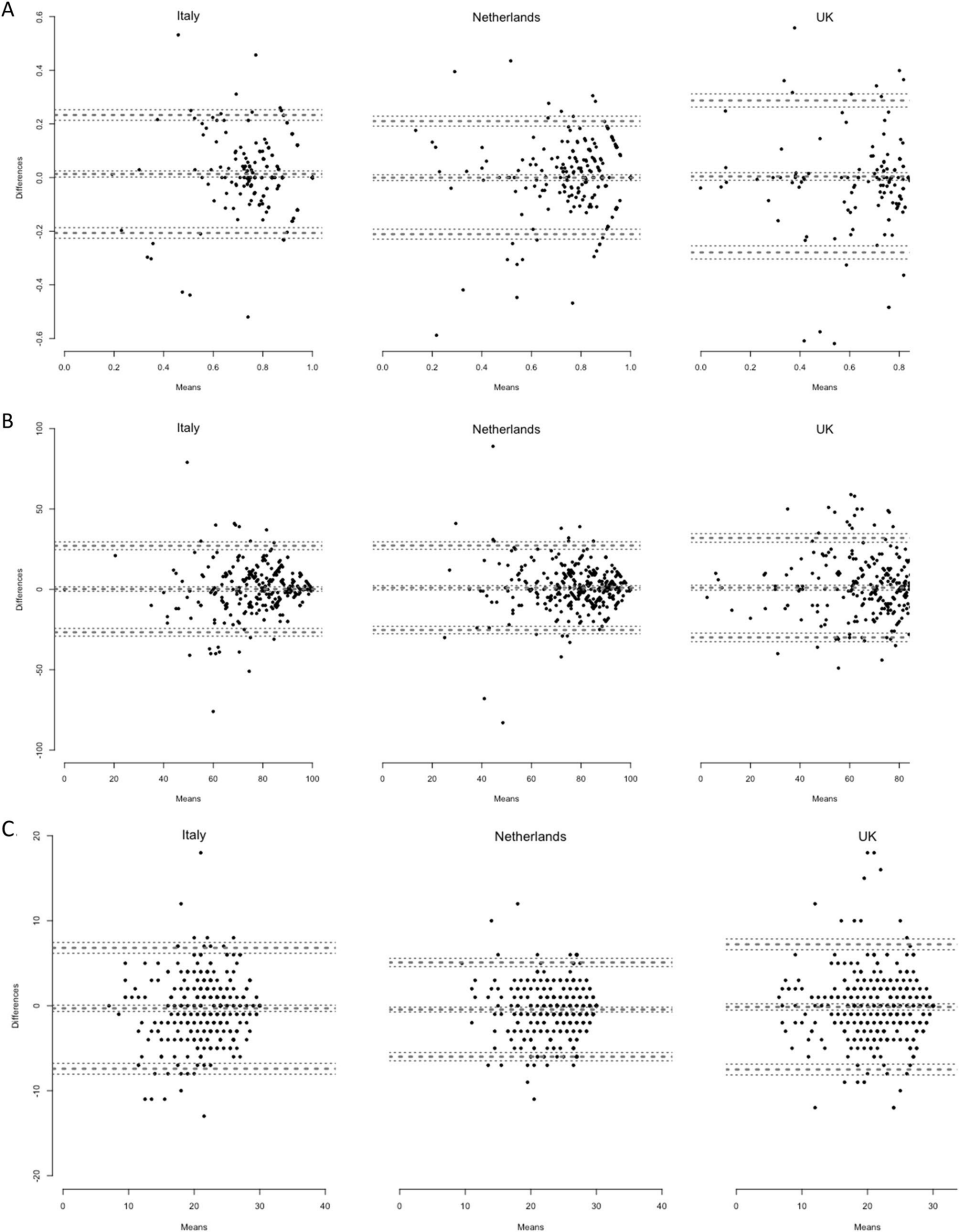
Bland–Altman plots of the EQ-5D-5L summary index **a** the EQ VAS (**b**), and the reworded QOLIBRI-OS level sum score **c** in Italy, the Netherlands, and the UK

BA plots for the EQ VAS, the EQ-5D-5L summary index, and reworded QOLIBRI-OS level sum score (Fig. 2) suggested no significant systematic differences between T1 and T2 and permit further examinations.

### Test–retest reliability

Table 2 presents the test–retest reliability results for the EQ-5D-5L dimensions, the EQ-5D-5L summary index, the EQ VAS, the reworded QOLIBRI-OS items, and the reworded QOLIBRI-OS level sum score.

**Table 2:** Gwet’s AC2, the percentage agreement of the EQ-5D-5L and the reworded QOLIBRI-OS; Intraclass correlation coefficient and concordance correlation coefficient of the EQ VAS, the EQ-5D-5L summary index, and the reworded QOLIBRI-OS level sum score in Italy, the Netherlands, and the UK

	Italy		Netherlands		UK	
	Gwet’s AC2 (95%CI)	% Agree.^a^	Gwet’s AC2 (95%CI)	% Agree	Gwet’s AC2 (95%CI)	% Agree
EQ-5D-5L						
Mobility	0.93 0.90–0.95)	90.7	0.81 (0.77–0.86)	80.8	0.88 (0.85–0.92)	86.6
Self-care	0.98 (0.97–0.99)	97.1	0.94 (0.92–0.96)	92.1	0.94 (0.92–0.96)	92.6
Usual activities	0.91 (0.88–0.94)	89.4	0.81 (0.76–0.85)	79.2	0.86 (0.82–0.89)	83.9
Pain/Discomfort	0.65 (0.59–0.71)	69.0	0.64 (0.58–0.71)	70.8	0.74 (0.70–0.79)	76.0
Anxiety/Depression	0.67 (0.61–0.72)	69.2	0.76 (0.72–0.81)	75.6	0.71 (0.66–0.77)	72.8

	ICC (trans.^b^) 95% CI	CCC 95% CI	ICC (trans.) 95% CI	CCC 95% CI	ICC (trans.) 95% CI	CCC 95% CI
EQ VAS	0.62 (0.56–0.68)	0.61 (0.54–0.67)	0.67 (0.61–0.72)	0.64 (0.58–0.70)	0.66 (0.60–0.71)	0.68 (0.63–0.73)
EQ-5D Summary index	0.76 (0.72–0.80)	0.76 (0.72–0.80)	0.81 (0.77–0.84)	0.81 (0.77–0.84)	0.85 (0.82–0.87)	0.84 (0.81–0.87)

	Gwet’s AC2 (95%CI)	% Agree	Gwet’s AC2 (95%CI)	% Agree	Gwet’s AC2 (95%CI)	% Agree
Reworded QOLIBRI-OS						
Physical condition	0.55 (0.49–0.62)	56.0	0.62 (0.56–0.67)	62.8	0.47 (0.40–0.53)	55.4
Cognition	0.57 (0.51–0.63)	58.4	0.63 (0.57–0.68)	62.8	0.51 (0.44–0.57)	56.2
Emotion	0.47 (0.41–0.54)	50.7	0.55 (0.49–0.60)	53.6	0.45 (0.38–0.51)	52.7
Function in daily activities	0.47 (0.47–0.54)	49.9	0.64 (0.58–0.70)	65.4	0.52 (0.46–0.58)	56.9
Personal and social life	0.45 (0.38–0.51)	50.4	0.59 (0.53–0.65)	59.7	0.41 (0.34–0.48)	53.0
Current situation and future prospects	0.39 (0.32–0.46)	46.7	0.54 (0.48–0.60)	58.2	0.44 (0.37–0.50)	53.0

	ICC (trans.) 95% CI	CCC 95% CI	ICC (trans.) 95% CI	CCC 95% CI	ICC (trans.) 95% CI	CCC 95% CI
Reworded QOLIBRI-OS level sum score	0.69 (0.64–0.74)	0.69 (0.64–0.74)	0.77 (0.73–0.81)	0.78 (0.74–0.81)	0.76 (0.72–0.80)	0.77 (0.73–0.81)

^a^Percentage (%) agreement

^b^Transformed using Tukey’s “Ladder of Powers”

Gwet’s AC2 for the EQ-5D-5L dimensions was the highest in Self-care (> 0.9) in all three countries, followed by Mobility and Usual activity in Italy, showing almost perfect reliability in these dimensions. Gwet’s AC2 in Pain/Discomfort and Anxiety/Depression were lowest, showing substantial reliability. The ICC and the CCC for the EQ-5D-5L summary index and the EQ VAS respectively are fairly similar. They range from 0.73 to 0.84 for the EQ-5D-5L summary index and 0.61 to 0.68 for EQ VAS, showing moderate to good reliability in the three countries.

Gwet’s AC2 for the reworded QOLIBRI-OS items were lower than Gwet’s AC2 for the EQ-5D-5L dimensions, ranging from 0.39 to 0.63, suggesting substantial to fair reliability in different items. The ICC and the CCC range from 0.69 to 0.78, showing moderate to good reliability in the three countries.

Between countries, there were significant differences in the test–retest reliability in most domains of EQ-5D-5L, EQ-5D-5L summary index, EQ VAS, and reworded QOLIBRI-OS. (Supplementary file 2).

## Discussion

We investigated the test–retest reliability of the EQ-5D-5L and the reworded QOLIBRI-OS in Italy, the Netherlands, and the UK. For all three countries, we found substantial to almost perfect test–retest reliability in the five EQ-5D-5L dimensions and good and moderate reliability in the summary index and EQ VAS, respectively. The test–retest reliability of the reworded QOLIBRI-OS was systematically lower, with moderate to fair test–retest reliability in all countries.

The difference in test–retest reliability between the EQ-5D-5L dimension and reworded QOLIBRI-OS items may be explained by the fact that these two instruments seem to measure different aspects of health. First of all, median reworded QOLIBRI-OS level sum scores were almost the same at T1 and T2, but more individual changes were present, which resulted in lower test–retest reliability. Secondly, even though there were strong correlations between some domains of the reworded QOLIBRI-OS and the EQ-5D-5L, large differences in the test–retest reliability were found in these domains. The differences listed above indicate that the reworded QOLIBRI-OS measure satisfaction with quality of life, thus are susceptible to rating errors such as central tendency error [31], and may be affected by factors such as mood [32]. Another issue of using the reworded QOLIBRI-OS in the general population is interpretability [33]. Assigning meaningful interpretations of the scores requires extra measures such as population norm scores. Such data have only been recently measured in a few countries. The reworded QOLIBRI-OS is essentially an instrument for satisfaction with quality of life and further research is needed to investigate the psychometric properties of the reworded QOLIBRI-OS used in the general population. Preceding studies have found higher test–retest reliability of the QOLIBRI-OS in TBI [34] and stroke [35] patients than in our study population. This may indicate that the (reworded) QOLIBRI-OS is more sensitive in the injured population than in the general population.

The finding of our study that the reworded QOLIBRI-OS showed more individual changes between T1 and T2 compared to the EQ-5D-5L, may also suggest that the reworded QOLIBRI-OS is more sensitive for changes in the health status of the general population, which cannot be measured with the chronic disease items but are important for self-perceived health.

The test–retest reliability of the EQ-5D-5L in the general population found in our study is comparable to the test–retest reliability reported in studies that used in-person interviews to administer the EQ-5D-5L. A study from Indonesia [10] also used Gwet’s AC to assess test–retest reliability and showed similar results as ours. A study from South Korea [9] measured the reliability of EQ-5D-5L in the general population using Cohen’s Kappa and ICC. It confirmed the ‘Kappa’s paradox’ phenomenon of high agreement with low Kappa coefficient and the results showed lower reliability than in our study.

### Strengths and limitations

A strength of our study was that we adopted Gwet’s AC to access test–retest reliability. Compared to the commonly used Kappa coefficient, Gwet’s AC does not depend on the assumption of normal distribution and independence between raters, and it adjusts the chance-agreement probability when trait distribution is unbalanced. It offers a better estimation of test–retest reliability in the general population where trait distribution is extremely unbalanced. A second strength was that we compared the test–retest reliability of EQ-5D-5L and reworded QOLIBRI-OS in the general population of three countries.

Our study has some limitations too. First, the 93 (8%) respondents that had non-corresponding answers on gender, age, and education between T1 and T2 and/or spent less than 1 min to finish the questionnaire were included in the analysis. Including these respondents in the study might have affected the reliability of the results, even though we found no significant difference in the distribution of their responses on the EQ-5D-5L, and reworded QOLIBRI-OS compared to the rest. Second, the data were collected through the internet. The disadvantages related to web-based questionnaires include sampling issues and response rate [36]. Thirdly, the time interval between T1 and T2 in our studies was six months, which could allow for true health changes to occur [37]. Moreover, season variations have an impact on physical and mental health of a population [38]. These factors may have affected the test–retest reliability of the EQ-5D-5L, and reworded QOLIBRI-OS found in our study, they could also explain the significant differences of test–retest reliability between countries. Two weeks is generally considered the most appropriate time interval to investigate test–retest reliability [39], but no evidence has been found against longer time intervals. We sampled from the general population which is generally healthy and stable, and we excluded respondents who experienced a change in chronic health status from T1 to T2. We, therefore, believe that the time interval used in our study does not jeopardize the results on test–retest reliability. Finally, the use of monetary incentives may decrease response bias by design, but it may also increase response bias when accepting an incentive is correlated with education [40] and race [41]. In our study, the sample at T1 is designed to represent the socio-demographic of the general population. We are thus less worried about the bias induced by monetary incentives.

It is also worth noticing that using the EQ-5D-5L summary index and the reworded QOLIBRI-OS level sum score comes with some limitations too. Firstly, we used the value set for EQ-5D-5L of the UK for Italy because that of Italy was not available, different results might be produced using a different value set. Secondly, the reworded QOLIBRI-OS level sum score summary score with equal weight of each item. The use of equal weights means that each dimension is given equal importance in value judgement, which is not necessarily the case in different populations. However, no value set is available for the reworded QOLIBRI-OS yet.

## Conclusions

Test–retest reliability of the EQ-5D-5L, administered via a web-based questionnaire in general population samples from Italy, the Netherlands, and the UK, was substantial to almost perfect for the EQ-5D-5L dimensions, good for EQ-5D-5L summary index, and moderate for the EQ VAS. However, test–retest reliability was less satisfactory for the reworded QOLIBRI-OS. This indicates that the web-based EQ-5D-5L is a reliable instrument for the general population, but further research of the reworded QOLIBRI-OS is required.

## Supplementary Information

Below is the link to the electronic supplementary material.

Supplementary material 1 (DOCX 54 kb)

## Data Availability

The dataset used and analyzed during the current study is available from the senior author on reasonable request.

## Code availability

Available on request.
