# Supplementary material for: Test–retest reliability of the EQ-5D-5L and the reworded QOLIBRI-OS in the general population of Italy, the Netherlands, and the United Kingdom
Source: Qual Life Res. 2021 Jun 1;30(10):2961–71. doi: 10.1007/s11136-021-02893-3 (PMC8481194; doi:10.1007/s11136-021-02893-3)
Supplement: Supplementary file 1 — Supplementary material 1 (DOCX 54 kb) [file 11136_2021_2893_MOESM1_ESM.docx]

## Title: Test-retest reliability of the EQ-5D-5L and the QOLIBRI-OS in the general population of Italy, the Netherlands and the United Kingdom

## Journal: Quality of Life Research

## Authors: Di Long^1^, Suzanne Polinder^1^, Gouke J. Bonsel^1,2^, Juanita A. Haagsma^1^

Erasmus MC, University Medical Center Rotterdam, Department of Public Health, Rotterdam, the Netherlands.

EuroQol Group Executive Office, Rotterdam, The Netherlands.

***Corresponding author**

D. Long

Erasmus MC, University Medical Center Rotterdam, Department of Public Health

P.O. Box 2040, 3000 CA Rotterdam

The Netherlands

Tel. 0031 1070 38914

Email: d.long@erasmusmc.nl

# Supplementary file 1.

We compared the distribution of EQ-5D-5L and QOLIBRI-OS of the respondents who filled out their basic information differently between T1 and T2 and those who didn’t. The distribution at T1 and T2 and showed in table 1-2. The results showed no significant between the two groups at both T1 and T2 in most dimensions(items). Only EQ VAS at T2 showed significant difference in distribution(p<0.05). Based on these results, we did not the respondents with errors from our analyses.

Table 1. Distribution of the EQ-5D-5L, QOLIBRI-OS between the respondents with errors and with no errors in the baseline information at T1.

|  |  |  | With errors  (N=93) | Without errors  (N=1078) | P value |
| --- | --- | --- | --- | --- | --- |
| EQ-5D-5L | | |  |  |  |
|  | Mobility | |  |  | 0.840 |
|  |  | No problems | 77 (82.8%) | 904 (83.9%) |  |
|  |  | Slight problems | 12 (12.9%) | 119 (11.0%) |  |
|  |  | Moderate problems | 4 (4.3%) | 37 (3.4%) |  |
|  |  | Severe problems | 0 (0.0%) | 14 (1.3%) |  |
|  |  | Unable | 0 (0.0%) | 4 (0.4%) |  |
|  | Self-care | |  |  | 0.884 |
|  |  | No problems | 88 (94.6%) | 1017 (94.3%) |  |
|  |  | Slight problems | 4 (4.3%) | 34 (3.2%) |  |
|  |  | Moderate problems | 1 (1.1%) | 18 (1.7%) |  |
|  |  | Severe problems | 0 (0.0%) | 5 (0.5%) |  |
|  |  | Unable | 0 (0.0%) | 4 (0.4%) |  |
|  | Usual activities | |  |  | 0.490 |
|  |  | No problems | 78 (83.9%) | 880 (81.6%) |  |
|  |  | Slight problems | 12 (12.9%) | 119 (11.0%) |  |
|  |  | Moderate problems | 3 (3.2%) | 58 (5.4%) |  |
|  |  | Severe problems | 0 (0.0%) | 17 (1.6%) |  |
|  |  | Unable | 0 (0.0%) | 4 (0.4%) |  |
|  | Pain/discomfort | |  |  | 0.978 |
|  |  | No problems | 54 (58.1%) | 637 (59.1%) |  |
|  |  | Slight problems | 29 (31.2%) | 303 (28.1%) |  |
|  |  | Moderate problems | 9 (9.7%) | 104 (9.6%) |  |
|  |  | Severe problems | 1 (1.1%) | 25 (2.3%) |  |
|  |  | Unable | 0 (0.0%) | 9 (0.8%) |  |
|  | Anxiety/depression | |  |  | 0.151 |
|  |  | No problems | 65 (69.9%) | 682 (63.3%) |  |
|  |  | Slight problems | 19 (20.4%) | 238 (22.1%) |  |
|  |  | Moderate problems | 7 (7.5%) | 98 (9.1%) |  |
|  |  | Severe problems | 0 (0.0%) | 30 (2.8%) |  |
|  |  | Unable | 2 (2.2%) | 30 (2.8%) |  |
|  | EQ VAS | |  |  | 0.986 |
|  |  | Median (IQR) | 80(19) | 80(20) |  |
| QOLIBRI-OS | | |  |  |  |
|  | Physical condition | |  |  | 0.643 |
|  |  | Not at all satisfied | 4 (4.3%) | 60 (5.6%) |  |
|  |  | Slightly satisfied | 11 (11.8%) | 111 (10.3%) |  |
|  |  | Moderately satisfied | 28 (30.1%) | 278 (25.8%) |  |
|  |  | Quite satisfied | 37 (39.8%) | 486 (45.1%) |  |
|  |  | Very satisfied | 13 (14.0%) | 143 (13.3%) |  |
|  | Brain | |  |  | 0.211 |
|  |  | Not at all satisfied | 2 (2.2%) | 22 (2.0%) |  |
|  |  | Slightly satisfied | 6 (6.5%) | 70 (6.5%) |  |
|  |  | Moderately satisfied | 20 (21.5%) | 191 (17.7%) |  |
|  |  | Quite satisfied | 44 (47.3%) | 480 (44.5%) |  |
|  |  | Very satisfied | 21 (22.6%) | 315 (29.2%) |  |
|  | Feeling, emotion | |  |  | 0.925 |
|  |  | Not at all satisfied | 3 (3.2%) | 44 (4.1%) |  |
|  |  | Slightly satisfied | 4 (4.3%) | 80 (7.4%) |  |
|  |  | Moderately satisfied | 24 (25.8%) | 223 (20.7%) |  |
|  |  | Quite satisfied | 42 (45.2%) | 483 (44.8%) |  |
|  |  | Very satisfied | 20 (21.5%) | 248 (23.0%) |  |
|  | Daily activities | |  |  | 0.448 |
|  |  | Not at all satisfied | 2 (2.2%) | 30 (2.8%) |  |
|  |  | Slightly satisfied | 3 (3.2%) | 65 (6.0%) |  |
|  |  | Moderately satisfied | 19 (20.4%) | 185 (17.2%) |  |
|  |  | Quite satisfied | 45 (48.4%) | 441 (40.9%) |  |
|  |  | Very satisfied | 24 (25.8%) | 357 (33.1%) |  |
|  | Personal life | |  |  | 0.417 |
|  |  | Not at all satisfied | 2 (2.2%) | 57 (5.3%) |  |
|  |  | Slightly satisfied | 7 (7.5%) | 91 (8.4%) |  |
|  |  | Moderately satisfied | 23 (24.7%) | 243 (22.5%) |  |
|  |  | Quite satisfied | 38 (40.9%) | 453 (42.0%) |  |
|  |  | Very satisfied | 23 (24.7%) | 234 (21.7%) |  |
|  | Current situation | |  |  | 0.438 |
|  |  | Not at all satisfied | 5 (5.4%) | 91 (8.4%) |  |
|  |  | Slightly satisfied | 9 (9.7%) | 103 (9.6%) |  |
|  |  | Moderately satisfied | 25 (26.9%) | 278 (25.8%) |  |
|  |  | Quite satisfied | 34 (36.6%) | 410 (38.0%) |  |
|  |  | Very satisfied | 20 (21.5%) | 196 (18.2%) |  |

Table 2. Distribution of the EQ-5D-5L, QOLIBRI-OS between the respondents with errors and with no errors in the baseline information at T2.

|  |  |  | With errors  (N=93) | Without errors  (N=1078) | P value |
| --- | --- | --- | --- | --- | --- |
| EQ-5D-5L | | |  |  |  |
|  | Mobility | |  |  | 0.532 |
|  |  | No problems | 79 (84.9%) | 895 (83.0%) |  |
|  |  | Slight problems | 11 (11.8%) | 104 (9.6%) |  |
|  |  | Moderate problems | 3 (3.2%) | 59 (5.5%) |  |
|  |  | Severe problems | 0 (0.0%) | 16 (1.5%) |  |
|  |  | Unable | 0 (0.0%) | 4 (0.4%) |  |
|  | Self-care | |  |  | 0.199 |
|  |  | No problems | 90 (96.8%) | 1008 (93.5%) |  |
|  |  | Slight problems | 3 (3.2%) | 39 (3.6%) |  |
|  |  | Moderate problems | 0 (0.0%) | 17 (1.6%) |  |
|  |  | Severe problems | 0 (0.0%) | 9 (0.8%) |  |
|  |  | Unable | 0 (0.0%) | 5 (0.5%) |  |
|  | Usual activities | |  |  | 0.061 |
|  |  | No problems | 83 (89.2%) | 887 (82.3%) |  |
|  |  | Slight problems | 9 (9.7%) | 103 (9.6%) |  |
|  |  | Moderate problems | 1 (1.1%) | 66 (6.1%) |  |
|  |  | Severe problems | 0 (0.0%) | 16 (1.5%) |  |
|  |  | Unable | 0 (0.0%) | 6 (0.6%) |  |
|  | Pain/discomfort | |  |  | 0.106 |
|  |  | No problems | 58 (62.4%) | 598 (55.5%) |  |
|  |  | Slight problems | 26 (28.0%) | 304 (28.2%) |  |
|  |  | Moderate problems | 9 (9.7%) | 143 (13.3%) |  |
|  |  | Severe problems | 0 (0.0%) | 22 (2.0%) |  |
|  |  | Unable | 0 (0.0%) | 11 (1.0%) |  |
|  | Anxiety/depression | |  |  | 0.051 |
|  |  | No problems | 68 (73.1%) | 683 (63.4%) |  |
|  |  | Slight problems | 16 (17.2%) | 222 (20.6%) |  |
|  |  | Moderate problems | 5 (5.4%) | 104 (9.6%) |  |
|  |  | Severe problems | 2 (2.2%) | 47 (4.4%) |  |
|  |  | Unable | 2 (2.2%) | 22 (2.0%) |  |
|  | EQ VAS | |  |  | 0.043 |
|  |  | Median (IQR) | 81(19) | 80(20) |  |
| QOLIBRI-OS | | |  |  |  |
|  | Physical condition | |  |  | 0.841 |
|  |  | Not at all satisfied | 2 (2.2%) | 54 (5.0%) |  |
|  |  | Slightly satisfied | 8 (8.6%) | 102 (9.5%) |  |
|  |  | Moderately satisfied | 29 (31.2%) | 280 (26.0%) |  |
|  |  | Quite satisfied | 40 (43.0%) | 489 (45.4%) |  |
|  |  | Very satisfied | 14 (15.1%) | 153 (14.2%) |  |
|  | Brain | |  |  | 0.536 |
|  |  | Not at all satisfied | 1 (1.1%) | 16 (1.5%) |  |
|  |  | Slightly satisfied | 6 (6.5%) | 54 (5.0%) |  |
|  |  | Moderately satisfied | 18 (19.4%) | 198 (18.4%) |  |
|  |  | Quite satisfied | 42 (45.2%) | 478 (44.3%) |  |
|  |  | Very satisfied | 26 (28.0%) | 332 (30.8%) |  |
|  | Feeling, emotion | |  |  | 0.878 |
|  |  | Not at all satisfied | 1 (1.1%) | 42 (3.9%) |  |
|  |  | Slightly satisfied | 7 (7.5%) | 69 (6.4%) |  |
|  |  | Moderately satisfied | 23 (24.7%) | 235 (21.8%) |  |
|  |  | Quite satisfied | 41 (44.1%) | 475 (44.1%) |  |
|  |  | Very satisfied | 21 (22.6%) | 257 (23.8%) |  |
|  | Daily activities | |  |  | 0.882 |
|  |  | Not at all satisfied | 0 (0.0%) | 21 (1.9%) |  |
|  |  | Slightly satisfied | 4 (4.3%) | 52 (4.8%) |  |
|  |  | Moderately satisfied | 18 (19.4%) | 150 (13.9%) |  |
|  |  | Quite satisfied | 36 (38.7%) | 452 (41.9%) |  |
|  |  | Very satisfied | 35 (37.6%) | 403 (37.4%) |  |
|  | Personal life | |  |  | 0.713 |
|  |  | Not at all satisfied | 1 (1.1%) | 64 (5.9%) |  |
|  |  | Slightly satisfied | 8 (8.6%) | 80 (7.4%) |  |
|  |  | Moderately satisfied | 22 (23.7%) | 244 (22.6%) |  |
|  |  | Quite satisfied | 41 (44.1%) | 426 (39.5%) |  |
|  |  | Very satisfied | 21 (22.6%) | 264 (24.5%) |  |
|  | Current situation | |  |  | 0.872 |
|  |  | Not at all satisfied | 5 (5.4%) | 86 (8.0%) |  |
|  |  | Slightly satisfied | 12 (12.9%) | 102 (9.5%) |  |
|  |  | Moderately satisfied | 21 (22.6%) | 278 (25.8%) |  |
|  |  | Quite satisfied | 39 (41.9%) | 413 (38.3%) |  |
|  |  | Very satisfied | 16 (17.2%) | 199 (18.5%) |  |

# Supplementary file 3.

To compare the test-retest reliability between countries, we perform a Z test.

First, coefficients $\mu_{i}$ (Gwet’s AC, ICC and CCC) are transformed to Z scores ($Z_{i}$) by Fisher r-to-z transformation. Then, Z test is performed using the formula:

$$(Z_{1} - Z_{2}) / \sqrt{1 / (N_{1}- 3) + 1 / (N_{2} - 3)}$$

Where $N_{i}$ = the size of the population.

Based on the value of $Z_{i}$, null hypnosis ($H_{0}$) is set as:

If $Z_{1}>Z_{2},$ $H_{0}:\mu_{1}\leq\mu_{2} , H_{1}:\mu_{1}> \mu_{2}$;

If $Z_{1}<Z_{2},$ $H_{0}:\mu_{1}\geq\mu_{2}, H_{1}:\mu_{1}< \mu_{2}$;

Significant level is set at 0.05, one-tailed Z value is 1.645 (upper-tailed) or -1.645 (lower-tailed).

|  |  | Z score | | | Test score | | | Meaning |
| --- | --- | --- | --- | --- | --- | --- | --- | --- |
|  |  | IT | NL | UK | $\boldsymbol{\mu}_{\boldsymbol{IT}}\boldsymbol{,}\boldsymbol{\mu}_{\boldsymbol{NL}}$ | $\boldsymbol{\mu}_{\boldsymbol{IT}}\boldsymbol{,}\boldsymbol{\mu}_{\boldsymbol{UK}}$ | $\boldsymbol{\mu}_{\boldsymbol{NL}}\boldsymbol{,}\boldsymbol{\mu}_{\boldsymbol{UK}}$ |  |
| EQ-5D | |  |  |  |  |  |  |  |
|  | Mobility | 1.624 | 1.131 | 1.391 | 6.787* | 3.235* | -3.643* | $\mu_{IT}>\mu_{UK}>\mu_{NL}$ |
|  | Self-care | 2.373 | 1.743 | 1.764 | 8.684* | 8.470* | -0.293 | $\mu_{IT}>\mu_{NL}$  $\mu_{IT}>\mu_{UK}$ |
|  | Usual activities | 1.533 | 1.116 | 1.291 | 5.761* | 3.372* | -2.460* | $\mu_{IT}>\mu_{UK}>\mu_{NL}$ |
|  | Pain/  Discomfort | 0.775 | 0.765 | 0.961 | 0.138 | -2.587* | -2.750* | $\mu_{IT}<\mu_{UK}$  $\mu_{NL}<\mu_{UK}$ |
|  | Anxiety/  Depression | 0.802 | 1.002 | 0.897 | -2.760* | -1.324 | 1.473 | $\mu_{IT}<\mu_{NL}$ |
| EQ VAS | |  |  |  |  |  |  |  |
|  | ICC | 0.728 | 0.805 | 0.786 | -1.063 | -0.800 | 0.274 | / |
|  | CCC | 0.706 | 0.761 | 0.831 | -0.757 | -1.738* | -0.983 | $\mu_{IT}<\mu_{UK}$ |
| EQ-5D summary index | | |  |  |  |  |  |  |
|  | ICC | 1.001 | 1.127 | 1.238 | -1.739* | -3.303* | -1.563 | $\mu_{IT}<\mu_{NL}$  $\mu_{IT}<\mu_{UK}$ |
|  | CCC | 1.001 | 1.126 | 1.238 | -1.720* | -3.302* | -1.580 | $\mu_{IT}<\mu_{NL}$  $\mu_{IT}<\mu_{UK}$ |
| QOLIBRI-OS | |  |  |  |  |  |  |  |
|  | Physical condition | 0.626 | 0.720 | 0.507 | -1.297 | 1.642 | 2.976* | $\mu_{nl}>\mu_{UK}$ |
|  | Brain | 0.651 | 0.741 | 0.557 | -1.236 | 1.313 | 2.583* | $\mu_{NL}>\mu_{UK}$ |
|  | Feeling, emotion | 0.514 | 0.612 | 0.482 | -1.343 | 0.443 | 1.814* | $\mu_{NL}>\mu_{UK}$ |
|  | Daily activities | 0.512 | 0.757 | 0.577 | -3.385* | -0.916 | 2.520* | $\mu_{IT}<\mu_{NL}$  $\mu_{NL}>\mu_{UK}$ |
|  | Personal life | 0.481 | 0.679 | 0.436 | -2.734* | 0.621 | 3.408* | $\mu_{IT}<\mu_{NL}$  $\mu_{NL}>\mu_{UK}$ |
|  | Current situation | 0.410 | 0.604 | 0.470 | -2.672* | -0.823 | 1.889* | $\mu_{IT}<\mu_{NL}$  $\mu_{NL}>\mu_{UK}$ |
| QOLIBRI-OS sum level score | | |  |  |  |  |  |  |
|  | ICC | 0.856 | 1.028 | 1.003 | -2.374* | -2.055* | 0.342 | $\mu_{IT}<\mu_{NL}$  $\mu_{IT}<\mu_{UK}$ |
|  | CCC | 0.857 | 1.042 | 1.027 | -2.545* | -2.355* | 0.213 | $\mu_{IT}<\mu_{NL}$  $\mu_{IT}<\mu_{UK}$ |

*are significant results where $H_{0}$ is rejected.

Based on the test results, we concluded that the reliability coefficients in most domains are significantly different between countries as shown in the last column.
